# Methods of Instruction in Medicine

**Published:** 1889-07

**Authors:** 


### Methods of Instruction in Medicine.

Dr. J. West Roosevelt speaks as follows in a paper on Medical Education, published in the New York *Medical Journal*, March 16, 1889: Preliminary education is beginning to receive some of the attention it deserves. It is well to consider what is the best training for one who intends to study medicine. Let me avoid starting the old discussion of the relative value of classics or mathematics. It seems to me that, if it really be necessary to "train the mind" by the study of some language in the irritating and complicated way so long the fashion among teachers of Latin and Greek, the German might be made quite as dull, quite as involved and complex as Latin. When the student finishes the "training process" he will end with a more or less confused notion of a language which he must use and can learn perhaps more easily because of the training. I am not speaking of the rational teaching of Latin and Greek, merely of the method of lifeless drill in grammar so long in vogue.

Let us suppose that a student has a fair general education; what does he need to prepare for medical studies? Surely the answer should be: Special training in some branch of science requiring observation, classification, and more or less delicate use of the hands and special senses. It does not make much difference what is selected—chemistry, botany, comparative anatomy, or any other. Whatever it be let the student learn it not from a book alone, but let him do practical work. This does not preclude a good education in other matters; in fact, it presupposes such an education.

Perhaps the greatest need in medical education is some method for teaching teachers. It is almost unavoidable that medical instructors should go to work in an amateurish sort of way. Teaching to them is a small part of their aim in life. Most men really are practitioners first and teachers afterward. Many seek to teach merely because they can thereby advance their private practice. This is natural, as instructors are poorly paid. Unfortunately many men teach without having the faintest idea of the difficulties of so doing.

It is not only necessary that a teacher should know his subject; he should also know precisely what he wishes to teach about it. He should appreciate the difficulties likely to trouble his pupils. He should cultivate the faculty of expressing himself clearly and concisely. Should be on the lookout for illustrations and similes. Above all, he should try to learn how to make his pupils think for themselves. As in all educational institutions, so in medical schools, the greatest object should be to show *how* work is done.

